# Balloon-Assisted Coils Embolization for Ophthalmic Segment Aneurysms of the Internal Carotid Artery

**DOI:** 10.3389/fneur.2021.658661

**Published:** 2021-04-15

**Authors:** Liang Chaohui, Zhang Guang Yu, Hou Kai

**Affiliations:** Department of Neurosurgery, The Second Hospital of Hebei Medical University, Shijiazhuang, China

**Keywords:** ophthalmic segment aneurysms, balloon assisted coils, small and medium type, effective, safe

## Abstract

**Objective:** To explore the role of balloon-assisted coils technique for ophthalmic segment aneurysms (OSAS).

**Methods:** Clinical data of 30 patients with OSAS were reviewed between December 2017 and December 2018. OSAS were defined as arising from the internal carotid artery (ICA), reaching from the distal dural ring to the origin of the posterior communicating artery. OSAS were classified into four types based on the angiographic findings. The balloon-assisted coils technique was used for the embolization of aneurysms. The duration of balloon inflation cycles, as well as difficulty and complications during the embolization procedure, were recorded. The immediate angiographic results were evaluated according to the Raymond scale. Clinical results were evaluated based on the MRS score. Follow-ups were performed at 18 months post-embolization by DSA or MRA at our institution.

**Results:** Thirty-two aneurysms in 30 patients were detected by digital subtraction angiography (DSA), which included 30 unruptured and two ruptured cases. The patients with ruptured aneurysms were grade II status according to the Hunt-Hess scale. Three cases were type A, nine cases were type B, 17 cases were type C, and three cases were type D. According to aneurysm size, there were 19 cases of small, 11 cases of medium, two cases of large aneurysm. Thirty-two aneurysms were successfully embolized in 30 patients by balloon-assisted coils technique. The ophthalmic artery could be protected by an engorged balloon in the procedure, especially for type A aneurysms. Considering that type D aneurysm arises from the side-wall of the artery and near to tortuous ICA siphon, the balloon catheter was inflated to stabilize the microcatheter allowing for overinflation when necessary. The average duration of balloon dilatation was 4 min, and the average time was 2.5 times. Raymond class was one in 28 aneurysms and two in four aneurysms according to the immediate post-embolization angiographic results. All the patients achieved good clinical effects, except for one patient who presented with brain ischemia resulting in dizziness and contralateral limb weakness for 10 h due to prolonged temporary clamping of the responsible ICA. The follow-up angiography results were satisfactory at 18 months post-embolization.

**Conclusion:** OSAS endovascular treatment with balloon-assisted coils has different advantages in a different classification. The technique is safe, effective, and relatively inexpensive, especially for small and medium OSAS.

## Introduction

Intracranial aneurysms are the most common causes of subarachnoid hemorrhage affecting humans, which leads to high mortality and morbidity rates (1). Ophthalmic segment aneurysms (OSAS) are defined as aneurysms arising from the internal carotid artery (ICA), reaching from the distal dural ring to the posterior communicating artery's origin. Many reports indicated slower growth and lower risk of rupture for OSAS as compared to other intra-dural aneurysms (2, 3). Kumon et al. (4) proposed the treatment of all unruptured asymptomatic OSAS to prevent lethal subarachnoid hemorrhage. In contrast, De Jesus et al. (5) suggested that only patients with life expectancy >10 years should be treated if the aneurysm was >4 mm.

The endovascular intervention has been the main choice for intracranial aneurysm treatment since the international subarachnoid aneurysm trial (ISAT). Thus far, for endovascular management of OSAS, stent-assisted coils and flow-diverting devices have been mainly reported (6, 7). However, coiling and stent release in the endovascular procedure is difficult due to the tortuous ICA siphon. It is also challenging to treat OSAS by surgical clipping due to complex adjacent anatomy, proximity to the optic chiasma, and the clinoid process. Visual morbidity and loss were encountered due to optic damage during the surgery (8–12). Interestingly, Alberto et al. (13) found no difference in clinical outcomes after endovascular coiling or surgical clipping for ruptured carotid ophthalmic aneurysms.

The endovascular interventional strategy includes sole coils embolization, balloon-assisted coils, stent-assisted coils, or flow diversion. Yet, high bleeding risk due to dual antiplatelet therapy during the perioperative period (14), in-stent thrombosis (15), and stent malposition (16) can lead to poor prognosis if the patient received stent-assisted coils embolization or flow diversion implant. In contrast, the balloon-assisted coils embolization technique does not require dual antiplatelet medication, has low thrombosis formation, and more dense packing in the aneurysm sac, as was first reported by Moret (17) in 1997. The aim of this study was to share our experiences with balloon-assisted coils embolization in 30 patients with OSAS.

## Materials and Methods

### Study Subjects

A total of 30 patients with 32 OSAS, who were admitted at the Second Hospital of Hebei Medical University between December 2017 and December 2018, were included in this study. The inclusion criteria were: patients with life expectancy >10 years; the aneurysm sac of ≥4 mm in size. The exclusion criteria were: dissection, fusiform aneurysm, and giant aneurysm; the aneurysm sac of <4 mm in size. All the patients were consecutively enrolled in this study and had no family medical history. The patients or their relatives signed the informed consent for the surgery. All the patients who died of diseases other than intracranial aneurysms or unexpected events were excluded from the analysis.

The study was approved by the ethics committee of the Second Hospital of Hebei Medical University (Shijiazhuang, China).

### Treatment Regimen

All the patients received balloon-assisted coils embolization for the aneurysms under general anesthesia. Transarterial embolization was accomplished with a standard Seldinger puncture through the right femoral artery. A 6-F guiding catheter (Cordis Corporation, Miami Lakes, FL, USA) was advanced over a 0.035-inch guidewire (Terumo Corporation, Tokyo, Japan) into the petrous segment of the ICA. Heparin was intravenously administered, first as a 3,000 U bolus, followed by infusions at 1,000 U/hour. The size of the aneurysm neck and dome were measured by three-dimensional rotational angiography. A “working projection” was selected in sequence. Hyperglide balloon (4 mm × 15 mm/20 mm, Covidien/ev3, Irvine, CA, USA) or Scepter balloon (Microvention Corporation, USA) was navigated with the guidewire (0.010 & 0.014, Covidien/ev3, Irvine, CA, USA) to cover the aneurysm neck. Then, a micro-catheter tip with a transcend 0.014 soft-tip guide microwire (Stryker Neurovascular, CA, USA) was assisted into the aneurysm sac. The balloon was selected according to the sizes of the parent artery. The first coil was deployed under the dilation of the balloon. A contrast media/saline mixture of 50:50 was used to inflate the balloon catheter with a 1cc syringe. The balloon was inflated while placing the coils into the aneurysm until the stable coil frame appeared. The coil stability and its relationship with the parent artery were carefully observed when the balloon was slowly released in case the coils protruded into the parent artery. The duration of balloon inflation cycles was limited to no more than 5 min at a time, alternating with periods of at least 1 min of balloon deflation. The patients' balloon inflation cycles, difficulty, and complications during the embolization procedure were recorded, respectively. The packing process was terminated when the aneurysm staining disappeared in digital subtraction angiography. The catheter was removed under the protection of the dilated balloon.

### Therapeutic Evaluation

After the procedure, multiple angiographic projections were obtained to assess the immediate embolization effect. The embolization effect was graded according to the Raymond-Roy occlusion classification scale (18). The postoperative MRS scores of patients were recorded.

### Follow-Up and Prognosis Evaluation

Follow-ups were performed at 18 months post-embolization by DSA or MRA at our center. Meanwhile, clinical results were evaluated according to the MRS scores.

## Results

### Patient Data

In total, there were 30 patients, 26 female and four male, with a mean age of 54.87 ± 9.67 years (range, 26–67 years). The aneurysms included 30 unruptured and two ruptured cases. CT scan showed subarachnoid hemorrhage in the latter cases. In the unruptured group, one patient had a chief complaint of visual disorders for eight months, while the other 27 cases were incidentally identified. The patients with ruptured aneurysms were grade II according to the Hunt-Hess scale. The status of all patients was evaluated on admission according to the modified ranking scale (MRS). The MRS score of two patients with ruptured aneurysms was one point, and for others was 0 point. The patients' data and aneurysm characteristics are shown in Table 1.

**Table 1:** Patients' data and aneurysm characteristics.

**Patient No**.	**Gender/age**	**Clinical symptom**	**History**	**R/L**	**Type**	**Size (N & D)** ** mm**	**Complication**	**Results** ** (Raymond)**	**MRS** ** (discharge)**	**18** ** months**
1	F/65	Incidental	Hp	R	C(N)	3 × 4	NO	1	0	Stable
2	F/26	Sudden headache	Prenant	R	C(N)	4 × 5	NO	1	1	Stable
3	F/56	Incidental	Hp	R	C(w)	6 × 9	NO	1	0	Stable
4	F/59	Incidental	Gu	R	B (w)	6 × 15	NO	1	0	Stable
5	F/59	Incidental	DB smoking	R	C(w)	6 × 8	NO	1	0	Stable
6	F/50	Incidental	—	L	C(w)	3 × 5	NO	2	0	Stable
7	F/64	Incidental	—	R	B(w)	3.5 × 5	NO	1	0	—
8	F/61	Incidental	Hp	L	C(w)	4 × 6	NO	1	0	Stable
9	F/36	Incidental	—	L	A(w)	3 × 4	NO	1	0	—
10	M/59	Incidental	Hp smoking	R	B(w)	4 × 6	NO	2	0	Recurrent
11	F/48	Incidental	—	R	D(N)	3 × 5	NO	1	0	Stable
12	F/53	Incidental	Hp & CAD	L	B(N)	3 × 4	NO	1	0	Stable
13	M/61	Incidental	Smoking	L	C(w)	5 × 6	NO	2	0	—
14	F/65	Incidental	Hp	R	B(w)	5 × 11	NO	1	0	Stable
15	M/49	Visual disorder	Smoking & Hp	R	B(w)	4 × 13	NO	1	0	Stable
16	F/57	Incidental	Hp	R	C(w)	5 × 8	NO	1	0	Stable
17	F/37	Incidental	—	L	D(w)	4 × 6	NO	1	0	Stable
18	F/65	Incidental	Hp & CAD smoking	R	C(w)	4 × 6	YES	1	0	—
19	F/46	Incidental	Hp	R	C(w)	4.5 × 6	NO	1	0	Stable
20	F/51	Sudden headache	Hp	L	A(w)	5 × 7	NO	1	0	Stable
21	F/39	Incidental	—	L	C(w)	5x6	NO	1	0	Stable
22	F/61	Incidental	—	b	B(w): C(N)	3 × 5 & 3 × 7	NO	1	0	Stable
23	F/50	Incidental	Graves	R	B(w)	4 × 5	NO	1	0	Stable
24	F/56	Incidental	—	R	D(w)	3.5 × 4	NO	1	0	Stable
25	F/67	Incidental	Hp	R	C(w)	6 × 10	NO	2	0	—
26	F/64	Incidental	DB	b	C(w)	4 × 8 & 3 × 4	NO	1	0	Stable
27	F/64	Incidental	Hp	R	B(w)	10 × 12	NO	1	0	Stable
28	F/60	Incidental	Hp	L	C(w)	6 × 9	NO	1	0	Stable
29	F/53	Incidental	—	L	C(w)	7 × 11	NO	1	0	Stable
30	M/53	Incidental	Hp & CAD	L	A(w)	4 × 6	NO	1	0	Stable

*Hp, hypertension; GU, gastric ulcer; DB, diabetes; CAD, coronary heart disease; b, bilateral; W, wide neck; N, narrow neck*.

### Aneurysm Characteristics

**Figure 1:**
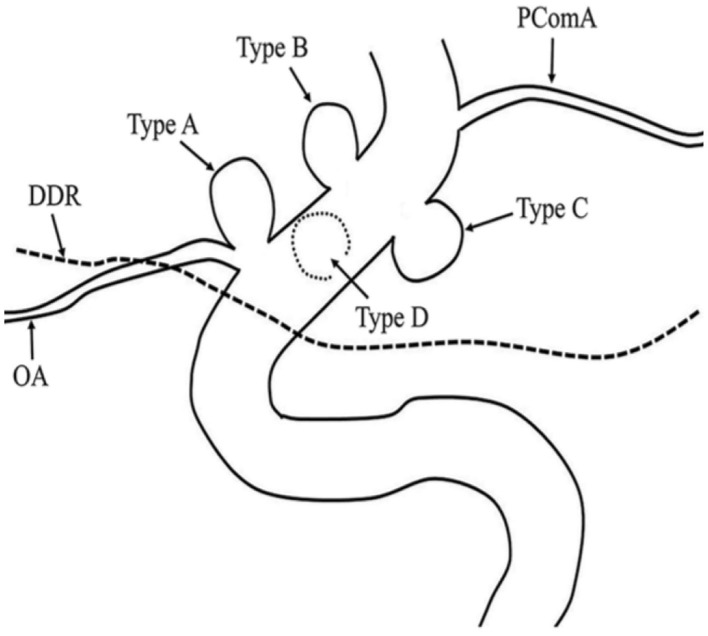
Ophthalmic segment aneurysm classification. Aneurysms arising from the superior ophthalmic segment of the internal carotid artery (ICA) with (Type A) and without (Type B) involvement of the ophthalmic artery. Aneurysms arising from the ventral (Type C) and medial (Type D) ophthalmic segment of the ICA.

OSAS were classified into four types based on the angiographic findings and endovascular considerations from previous reports (19). The types of aneurysms in this study are shown in Figure 1. Three cases were type A, nine cases were type B, 17 cases were type C, and three cases were type D. The neck of the aneurysm was manually measured based on DSA. Aneurysms were considered as wide-necked if the neck was ≥4 mm or the aneurysm had a dome-to-neck ratio <2. There were 27 cases of wide-necked aneurysms and five cases of narrow-necked aneurysms. The size of the dome was classified based on the International Study of Unruptured Intracranial Aneurysms (ISUIA) (2) standard into <7 mm (small), 7–12 mm (medium), 13–24 mm (large), and >25 mm (giant). There were 19 cases of small, 11 cases of medium, and two cases of large aneurysms.

### Clinical Status of the Patients

Thirty-two aneurysms were successfully treated with the balloon-assisted coils technique. The average duration of balloon dilatation was 4 min, and the average time was 2.5 times. One patient (case 18) developed procedure-related complications. She presented with transient brain ischemia resulting in dizziness and contralateral limb weakness lasting for 10 h due to prolonged temporary clamping of the responsible ICA (balloon inflation time, in this case, was 7 and 3 min, respectively). The patient with visual disorders (case 15) showed no aggravation post-procedure. No persistent complications occurred in this group. MRS scores were 0 in all the cases, except for one case (case 2, MRS 1) at discharge.

### Immediate Angiography Results and Follow-Up

According to the immediate post-embolization angiographic results, Raymond class 1 was found in 28 aneurysms (total aneurysm occlusion 87.5%), while Raymond class 2 was found in four aneurysms (partial aneurysm occlusion 12.5%). Twenty-five patients received angiography during the follow-up at 18 months post-embolization. Five patients were lost to follow-up. Twenty-four patients showed stable results, while one patient had aneurysm neck residue (case 10) and further stent cover at the 18 months follow-up. No new bleeding occurred during the follow-up period. The patients' data, clinical symptoms, aneurysm characteristics, immediate embolization outcomes, and follow-up results are presented in Table 1.

### Case Illustrations

**Figure 2:**
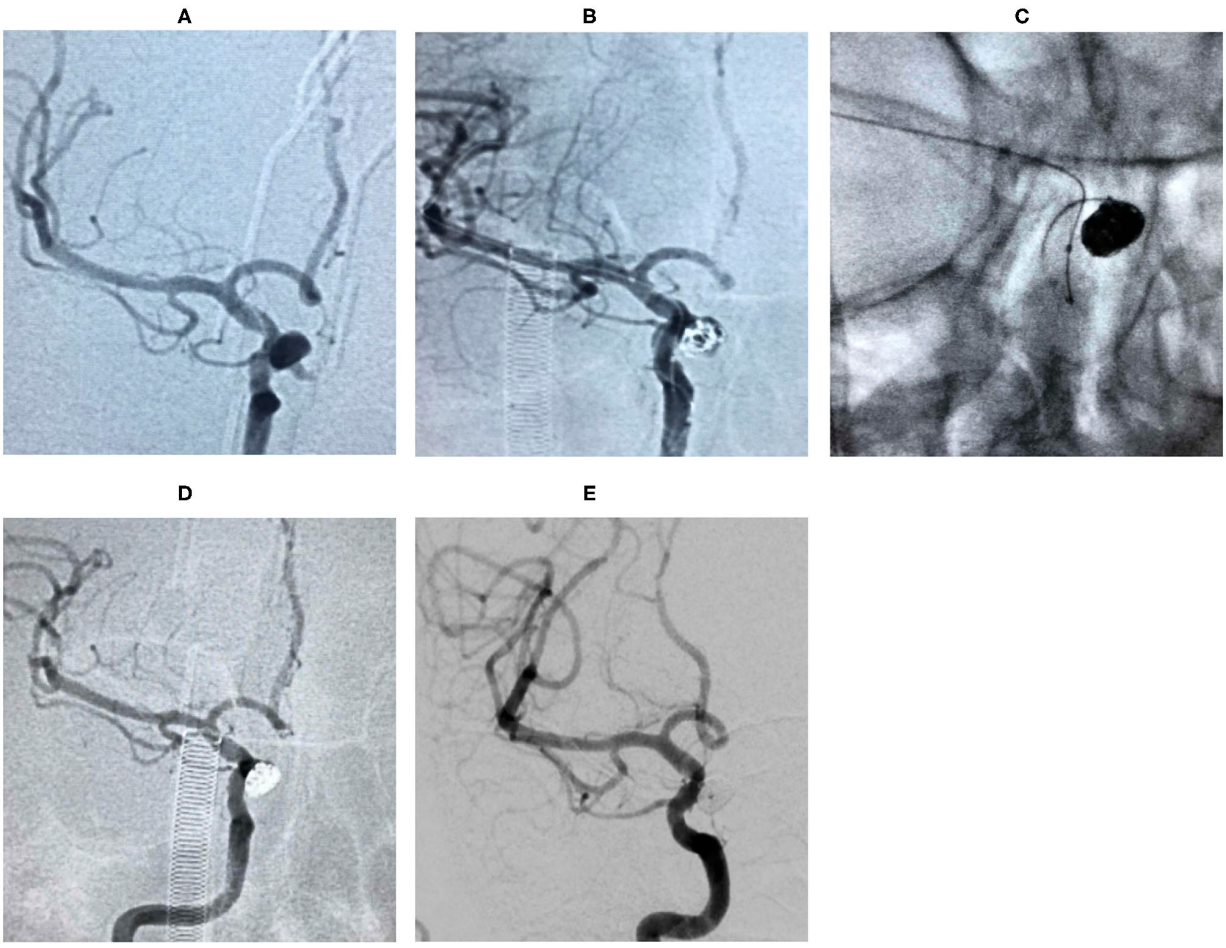
Digital subtraction angiography (DSA) showed that the aneurysm originated from the right ophthalmic segment of ICA **(A)**. The aneurysm was treated during the procedure **(B,C)**. The lesion was completely embolized in the immediate angiographic results **(D)** and 18 months follow-up **(E)**.

**Figure 3:**
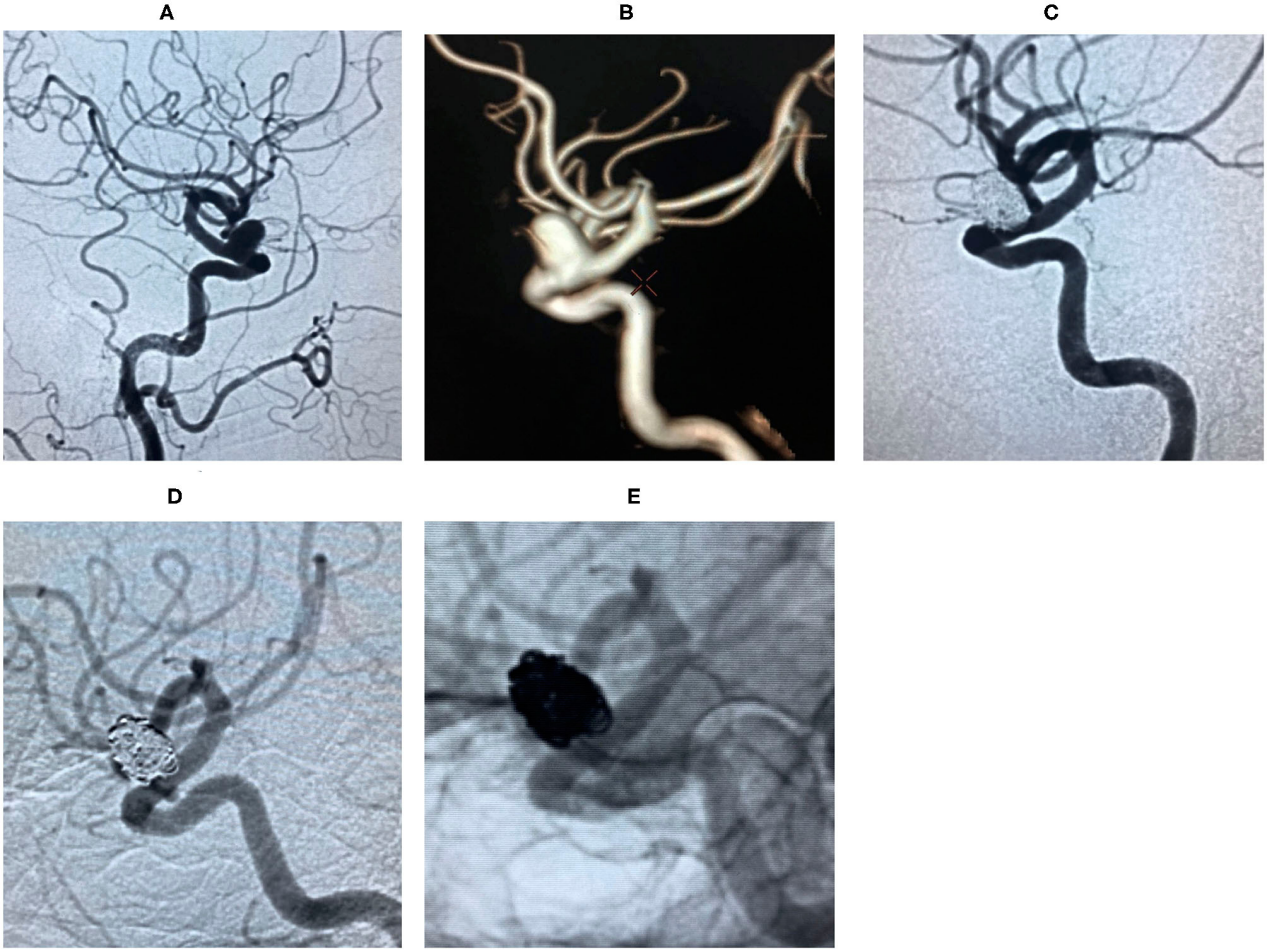
Digital subtraction angiography (DSA) revealed right ophthalmic segment aneurysm ~15 mm wide, with a neck size of 6 mm **(A,B)**. The aneurysm was completely embolized by balloon-assisted coils technique and the immediate post-embolization angiographic result is shown in **(C)**. The lesion was stable by DSA at the 18 months follow-up **(D,E)**.

**Figure 4:**
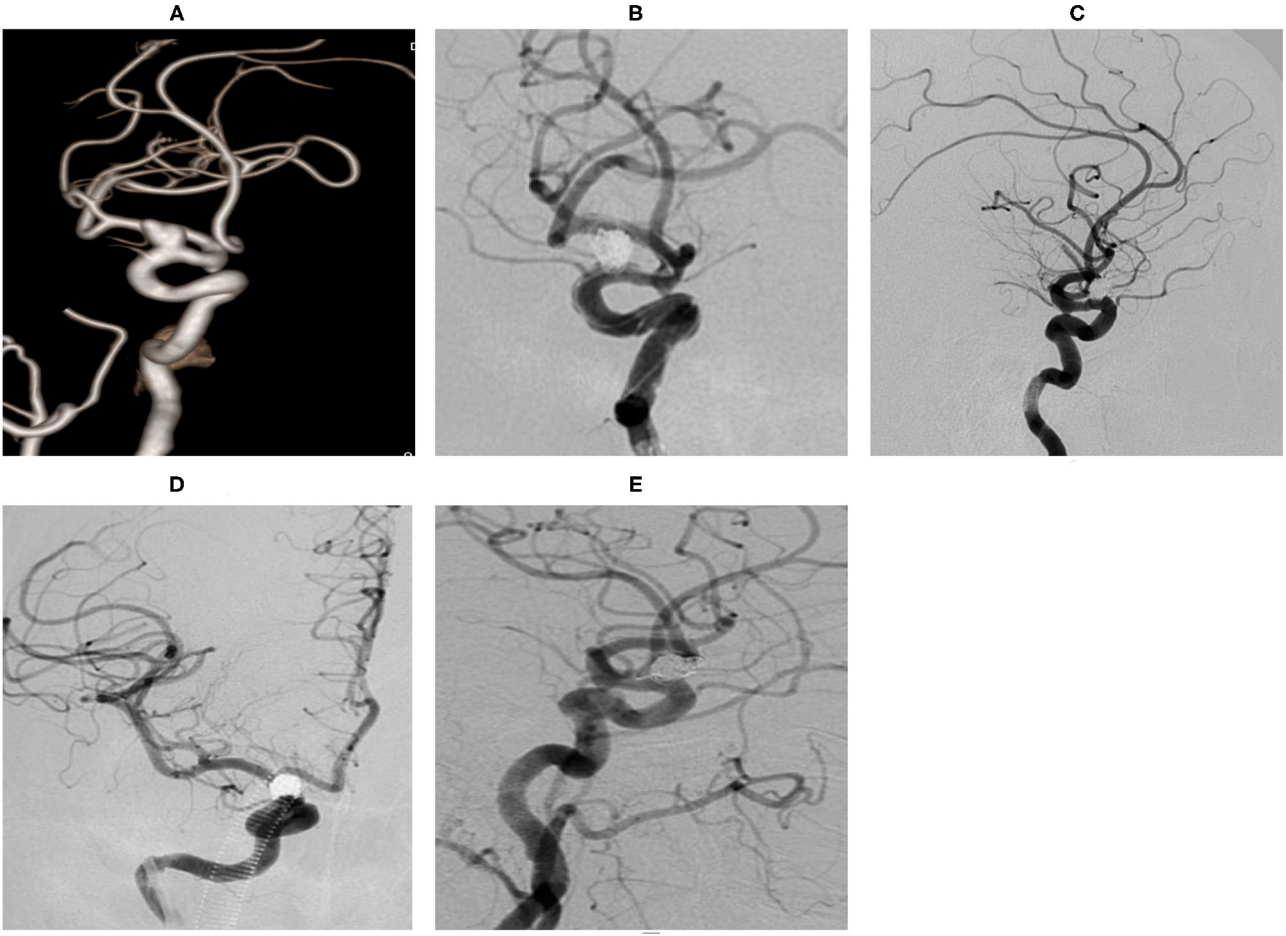
An ophthalmic segment aneurysm ~11 mm wide, with a neck size of 5 mm was detected by angiography **(A)**. A protective balloon covered the aneurysm neck in the procedure **(B)**. The aneurysm was completely embolized and the immediate post-embolization angiographic result is shown in **(C)**. The aneurysm had disappeared at the 18 months follow-up by DSA **(D,E)**.

**Figure 5:**
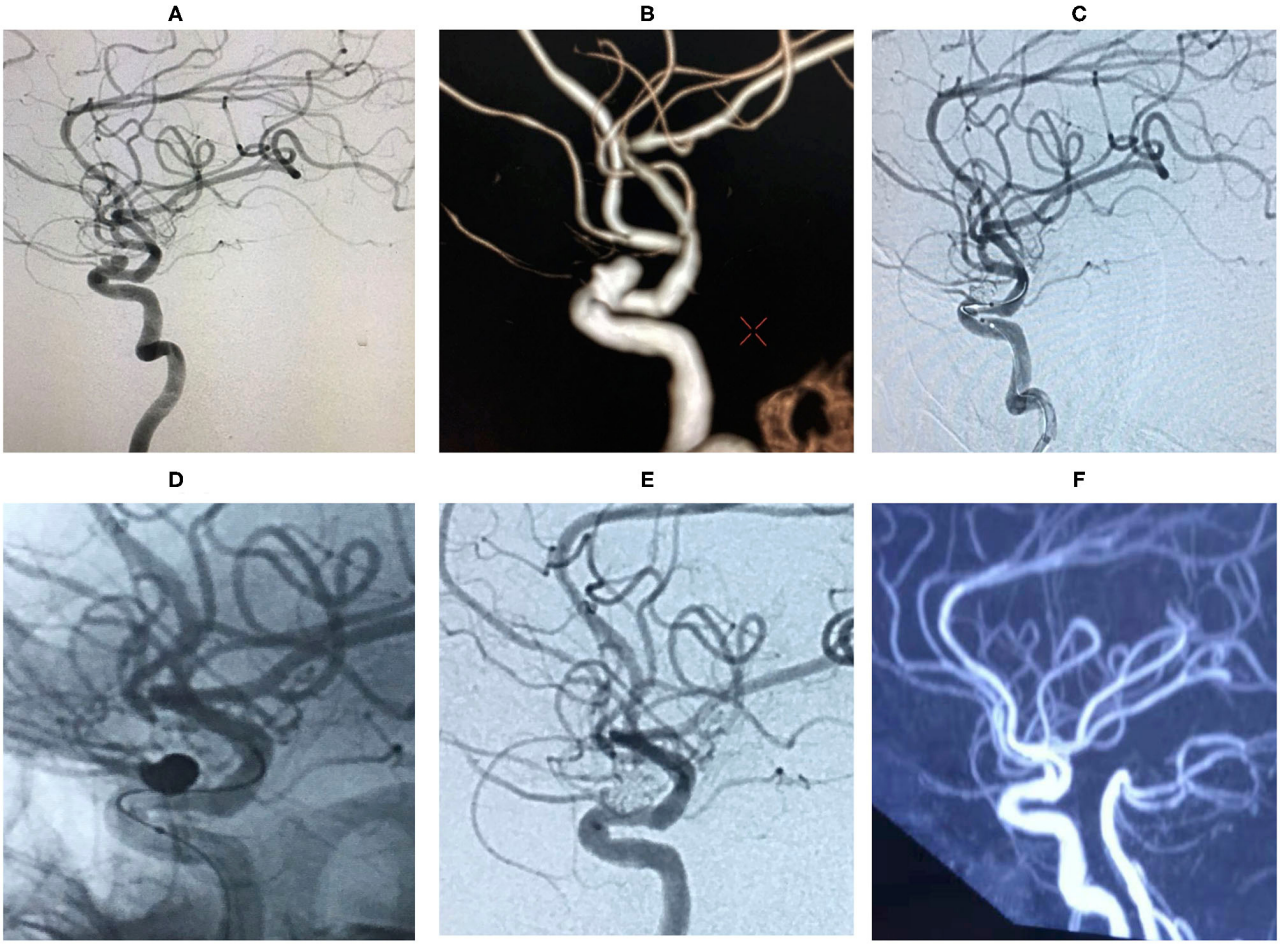
An ophthalmic segment irregular aneurysm ~7 mm wide, with a neck size of 5 mm was detected by angiography **(A,B)**. A protective balloon covered the aneurysm neck in the procedure **(C,D)**. The aneurysm was completely embolized and ophthalmic artery patency, and the immediate post-embolization angiographic result is shown in **(E)**. The aneurysm had disappeared at the 18 months follow-up by Magnetic Resonance angiography (MRA) **(F)**.

**Figure 6:**
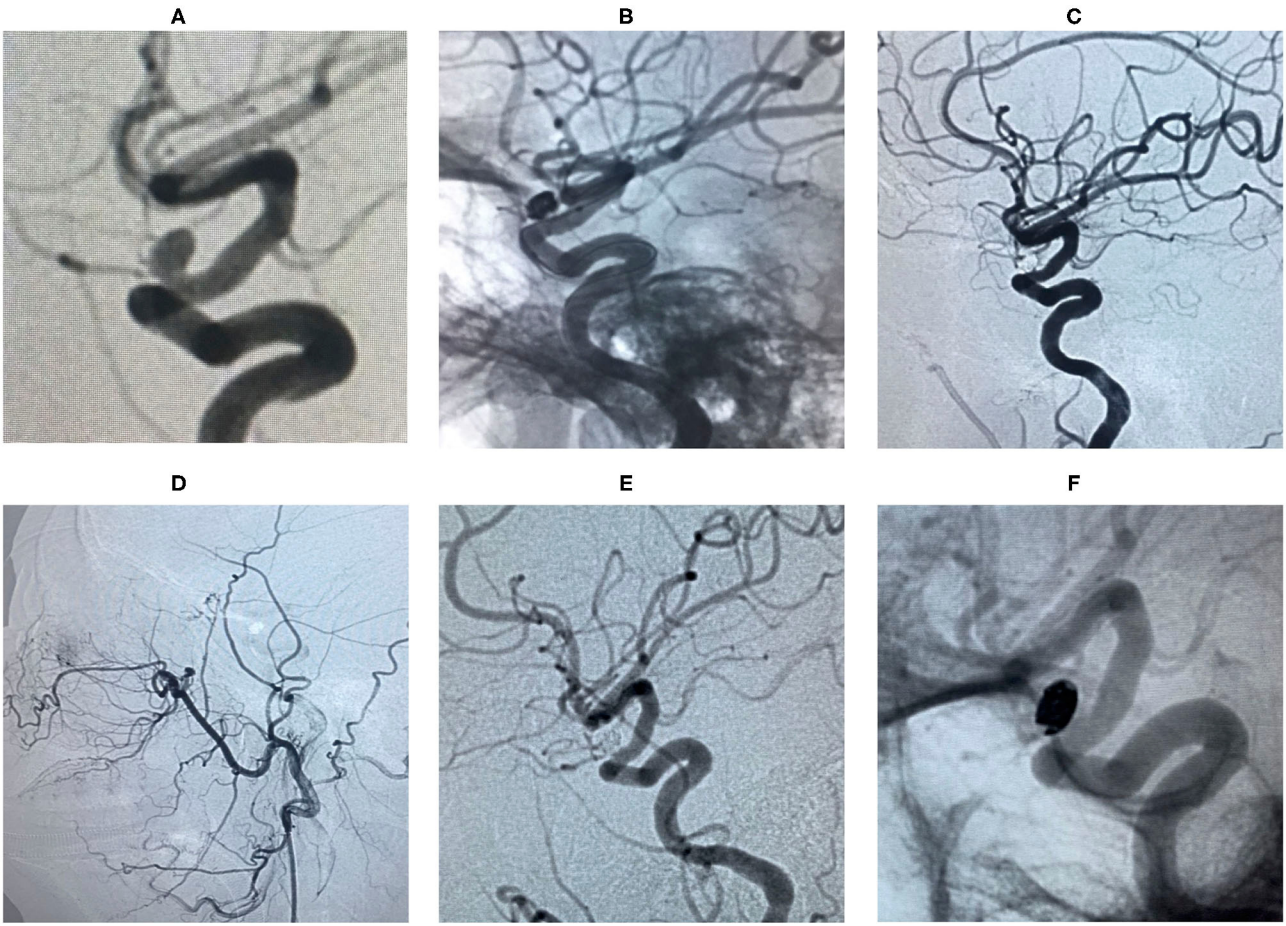
An ophthalmic segment aneurysm ~6 mm wide, with a neck size of 4 mm was detected by angiography **(A)**. A protective balloon covered the aneurysm neck in the procedure **(B)**. The aneurysm was embolized and the immediate post-embolization angiographic result is shown in **(C)**. The patient has favorable collaterals in **(D)** and no visual disorders. The aneurysm had disappeared completely and ophthalmic artery patency at the 18 months follow-up by DSA **(E,F)**.

**Case 3**. During the physical examination, magnetic resonance angiography (MRA) detected an intracranial aneurysm in a 56-year-old female patient (Figure 2).
**Case 4**. During a physical examination, MRA detected an intracranial aneurysm for 1 month in a 59-year-old male. He suffered from gastric ulcer for 1 year (Figure 3).
**Case 14**. MRA detected an intracranial aneurysm for 1 week in a 65-year-old female (Figure 4).
**Case 20**. A 51-year-old female presented a sudden headache lasting for 2 days. CT scan showed subarachnoid hemorrhage (Figure 5).
**Case 30**. A 53-year-old male was found to have intracranial aneurysms for 1 month by magnetic resonance angiography (MRA) (Figure 6).

## Discussion

Ophthalmic segment aneurysms (OSAS) are defined as originating from the distal ring to post communication artery of the internal carotid artery (ICA). Gross (20) reported a higher risk of rupture, large size, aneurysm growth, and aneurysm irregularity for OSAS. However, other reports indicated slower growth and lower risk of rupture for OSAS as compared to other intra-dural aneurysms (2, 3). In this study, there were 30 unruptured and two ruptured aneurysms (4 × 5 mm and 4 × 6 mm in size, respectively). This study revealed that OSAS had lower rupture rates than other intracranial aneurysms.

In this study, satisfactory clinical results were obtained for OSAS patients with balloon-assisted coils technique. There were no deaths in this study. Only one patient presented with procedure-related complication in the form of transient brain ischemia, which resulted in dizziness and contralateral limb weakness for 10 h due to prolonged temporary clamping of the responsible ICA. Yet, due to the symptoms, it was not possible to exclude the tiny cerebral infarction caused by small atherosclerotic plaque falling off caused by balloon inflation. His symptoms were gradually relieved, and he completely recovered after the 10 h long procedure and no MRI scanning. Yadla et al. (21) reported 1.4% morbidity and 0% mortality in their study of 170 unruptured OSAS. Similarly, this study also achieved safe embolization and a good clinical prognosis.

The immediate angiography and 18 months follow-up results were also satisfactory. The complete occlusion incidence was 87.5% (28/32), and partial occlusion was seen in four cases (cases 6, 10, 13, and 25) in the angiographic results immediately post-embolization. Furthermore, only one aneurysm showed neck recurrence during the 18 months follow-up. Larger and wide-neck aneurysms have a higher recurrence rate (22). Flow-diversion is popular due to the high obliteration rate and low complication rate, and high rate of improvement of visual symptoms (23). The pipeline embolization device (PED) achieved higher complete occlusion than stent-coiling in cases with larger aneurysms (24–26). Still, visual blurring and visual field defects occur after PED treatment (27–30). The visual impairment may result from the direct mass effect of the aneurysm sac compressing the optic nerve, inflammation, or retinal artery thrombosis (27). Also, the rate of visual complications for type A aneurysms was higher than in other types (31). In this study, there were three cases of type A aneurysm. The ophthalmic artery could be protected by an engorged balloon, especially for type A aneurysms. The cases with visual symptoms (case 15) did not aggravate after the procedure because the aneurysm size was relatively small. In this study, there were only two large aneurysms (6 × 15 mm, 4 × 13 mm) treated by the balloon-assisted coils technique. Both patients achieved satisfactory angiographic results during the 18 months follow-up. Furthermore, the balloon-assisted coils technique is inexpensive as compared to PED in China. However, larger prospective studies with longer follow-up are warranted in the future, especially for large aneurysms.

The balloon-assisted coils technique has an important role in the aneurysm neck remodeling during the embolization procedure. In addition to the previously published advantages (17, 32, 33), we observed that the first coil could easily form the steady frame and conveniently achieve further compact embolization once the blood flow was temporarily blocked. The necks of aneurysms were remodeled by an engorged balloon. Thereafter, the coils formed a saddle structure to decrease the recurrence of the aneurysm. Second, type D aneurysm originated from the side wall of the vessels and near to the tortuous ICA siphon. In our experience, it is more difficult to navigate the microcatheter tip into an aneurysm sac for type D aneurysm than type A, type B, and type C ones.

The balloon catheter was inflated to stabilize the coil mass or the microcatheter allowing for overinflation when necessary. Third, the dual-lumen Scepter balloon catheters provide some advantages compared to single-lumen Hyperglide balloon catheters in the operation process. The Scepter balloon catheter is compatible with 0.014-inch microguidewires, and 0.010-inch microguidewires for Hyperglide balloon catheters. Distal tip length is 5 mm for the Scepter, 4 mm for the Hyperglide. Therefore, the Scepter balloon catheter can be used to improve trackability and stability during the embolization procedure.

Balloon-assisted coiling potentially causes significant technical stress for aneurysm and parent vessel. Stents have been increasingly used for the treatment of wide-necked or complex aneurysms with various deployment techniques. Nevertheless, deployment tends to be difficult, and the stents kink or easily twist in the case of ICA siphon. Thrombosis forms in the stent in the case of anti-platelet drug resistance and defective wall attachment of the stent (34, 35). Prolonged bleeding risks may be caused by drugs if the patient received stent-assisted coils embolization. Nishido et al. (36) reported 7.0% ischemic and 2.3% hemorrhagic complications, with a 2.7% overall rate of procedure-induced mortality with stent-assisted coils technique.

Thromboembolic events were reported to be induced by the balloon-assisted coils technique during the aneurysm embolization (37–39). It is important to select the fitting size balloon for the prevention of thromboembolic complications. The under-sized balloon was unstable during inflation and showed a tendency to jump forward, inducing thromboembolic complications in the tortuous ICA siphon. Therefore, longer balloons had a more stable position during inflation and were particularly useful in a very wide-necked aneurysm. This study showed no permanent ischemia, even in patients with brain infarction. The patients with unruptured aneurysms were given 100 mg aspirin and 75 mg clopidogrel for 3 days before the procedure to reduce the ischemia events. Meanwhile, the balloon inflation time and frequency were also reduced during the operation to decrease the incidence of brain ischemia.

In summary, OSAS endovascular treatment with balloon-assisted coils has different advantages in a different classification. This is a safe, effective, and economical technique for treating OSAS, especially for small and medium aneurysms. Long-term follow-up is needed to ensure stable effects.

## Data Availability Statement

The original contributions presented in the study are included in the article/supplementary material, further inquiries can be directed to the corresponding author/s.

## Ethics Statement

The studies involving human participants were reviewed and approved by the ethics committee of the Second Hospital of Hebei Medical University (Shijiazhuang, China). The patients/participants provided their written informed consent to participate in this study.

## Author Contributions

LC: major in patients operation and management, follow-up, and write paper. ZY and HK: major in patients operation, management, and follow-up. All authors contributed to the article and approved the submitted version.

## Conflict of Interest

The authors declare that the research was conducted in the absence of any commercial or financial relationships that could be construed as a potential conflict of interest.
